# Intravitreal Gene Therapy vs. Natural History in Patients With Leber Hereditary Optic Neuropathy Carrying the m.11778G>A *ND4* Mutation: Systematic Review and Indirect Comparison

**DOI:** 10.3389/fneur.2021.662838

**Published:** 2021-05-24

**Authors:** Nancy J. Newman, Patrick Yu-Wai-Man, Valerio Carelli, Valerie Biousse, Mark L. Moster, Catherine Vignal-Clermont, Robert C. Sergott, Thomas Klopstock, Alfredo A. Sadun, Jean-François Girmens, Chiara La Morgia, Adam A. DeBusk, Neringa Jurkute, Claudia Priglinger, Rustum Karanjia, Constant Josse, Julie Salzmann, François Montestruc, Michel Roux, Magali Taiel, José-Alain Sahel

**Affiliations:** ^1^Departments of Ophthalmology, Neurology and Neurological Surgery, Emory University School of Medicine, Atlanta, GA, United States; ^2^Cambridge Centre for Brain Repair and MRC Mitochondrial Biology Unit, Department of Clinical Neurosciences, University of Cambridge, Cambridge, United Kingdom; ^3^Cambridge Eye Unit, Addenbrooke's Hospital, Cambridge University Hospitals, Cambridge, United Kingdom; ^4^Moorfields Eye Hospital National Health Service Foundation Trust, London, United Kingdom; ^5^UCL Institute of Ophthalmology, University College London, London, United Kingdom; ^6^Istituto di Ricovero e Cura a Carattere Scientifico Istituto delle Scienze Neurologiche di Bologna, Unitá Operativa Compless Clinica Neurologica, Bologna, Italy; ^7^Unit of Neurology, Department of Biomedical and Neuromotor Sciences, University of Bologna, Bologna, Italy; ^8^Departments of Neurology and Ophthalmology, Wills Eye Hospital and Thomas Jefferson University, Philadelphia, PA, United States; ^9^Department of Neuro Ophthalmology and Emergencies, A. de Rothschild Foundation Hospital, Paris, France; ^10^Centre d'investigation Clinique, Centre Hospitalier National d'Ophtalmologie des Quinze Vingts, Paris, France; ^11^Department of Neurology, Friedrich-Baur-Institute, University Hospital, Ludwig-Maximilians-University Munich, Munich, Germany; ^12^German Center for Neurodegenerative Diseases, Munich, Germany; ^13^Munich Cluster for Systems Neurology, Munich, Germany; ^14^Doheny Eye Institute, School of Medicine, University of California, Los Angeles, Los Angeles, CA, United States; ^15^Department of Ophthalmology, University Hospital, Ludwig-Maximilians-University Munich, Munich, Germany; ^16^Department of Ophthalmology, University of Ottawa Eye, Ottawa, ON, Canada; ^17^eXYSTAT, Data Management and Statistic, Malakoff, France; ^18^Medical and Regulatory Consulting, Paris, France; ^19^GenSight Biologics, Paris, France; ^20^Sorbonne Université, INSERM, CNRS, Institut de la Vision, Paris, France; ^21^A. de Rothschild Foundation Hospital, Paris, France; ^22^Department of Ophthalmology, The University of Pittsburgh School of Medicine, Pittsburgh, PA, United States; ^23^CHNO des Quinze-Vingts, Institut Hospitalo-Universitaire FOReSIGHT, INSERM-DGOS CIC 1423, Paris, France

**Keywords:** Leber hereditary optic neuropathy, *ND4*, gene therapy, natural history, visual acuity

## Abstract

**Objective:** This work aimed to compare the evolution of visual outcomes in Leber hereditary optic neuropathy (LHON) patients treated with intravitreal gene therapy to the spontaneous evolution in prior natural history (NH) studies.

**Design:** A combined analysis of two phase three randomized, double-masked, sham-controlled studies (REVERSE and RESCUE) and their joint long-term extension trial (CLIN06) evaluated the efficacy of rAAV2/2-*ND4* vs. 11 pooled NH studies used as an external control.

**Subjects:** The LHON subjects carried the m.11778G>A *ND4* mutation and were aged ≥15 years at onset of vision loss.

**Methods:** A total of 76 subjects received a single intravitreal rAAV2/2-*ND4* injection in one eye and sham injection in the fellow eye within 1 year after vision loss in REVERSE and RESCUE. Both eyes were considered as treated due to the rAAV2/2-*ND4* treatment efficacy observed in the contralateral eyes. Best corrected visual acuity (BCVA) from REVERSE, RESCUE, and CLIN06 up to 4.3 years after vision loss was compared to the visual acuity of 208 NH subjects matched for age and *ND4* genotype. The NH subjects were from a LHON registry (REALITY) and from 10 NH studies. A locally estimated scatterplot smoothing (LOESS), non-parametric, local regression model was used to modelize visual acuity curves over time, and linear mixed model was used for statistical inferences.

**Main Outcome Measures:** The main outcome measure was evolution of visual acuity from 12 months after vision loss, when REVERSE and RESCUE patients had been treated with rAAV2/2-*ND4*.

**Results:** The LOESS curves showed that the BCVA of the treated patients progressively improved from month 12 to 52 after vision loss. At month 48, there was a statistically and clinically relevant difference in visual acuity of −0.33 logarithm of the minimal angle of resolution (LogMAR) (16.5 ETDRS letters equivalent) in favor of treated eyes vs. NH eyes (*p* < 0.01). Most treated eyes (88.7%) were on-chart at month 48 as compared to 48.1% of the NH eyes (*p* < 0.01). The treatment effect at last observation remained statistically and clinically significant when adjusted for age and duration of follow-up (−0.32 LogMAR, *p* < 0.0001).

**Conclusions:** The m.11778G>A LHON patients treated with rAAV2/2-*ND4* exhibited an improvement of visual acuity over more than 4 years after vision loss to a degree not demonstrated in NH studies.

**Clinical Trial Registration:** NCT02652767, NCT02652780, NCT03406104, and NCT03295071.

## Introduction

Leber hereditary optic neuropathy (LHON) is a rare genetic disease caused by mutations of mitochondrial genes of the respiratory chain complex I, leading to selective degeneration of retinal ganglion cells and optic nerve atrophy (1). The disease typically manifests as severe central visual loss in one eye, followed by second eye involvement with a median interval of 8 weeks (2–4). The decline in visual acuity is subacute to rapidly progressive, with visual acuity usually deteriorating to values worse than 20/200 over a few months after onset (2, 5). Such sudden and profound vision loss occurring in well-sighted individuals, usually young adults, has a dramatic impact on their quality of life (5). Thus, targeted drug discovery and new therapeutic approaches are crucial to improve the visual prognosis of patients with LHON. While the oral drug idebenone has shown some benefit (6–8), leading to its approval for the treatment of LHON in Europe, there is still a pressing medical need for further therapies with a significant therapeutic benefit in LHON (7).

Among the three most common point mutations found in LHON, the m.11778G>A mutation in the mitochondrial *ND4* gene is the most prevalent, accounting for ~70% of LHON patients worldwide (9). Moreover, it is associated with a poor prognosis, with spontaneous visual recovery limited to <15% of patients, as shown by a recent meta-analysis of 695 patients with the m.11778G>A mutation (10).

rAAV2/2-*ND4* (also called lenadogene nolparvovec) is a replication-defective, recombinant adeno-associated virus vector serotype 2 (rAAV2) containing a codon-modified complementary DNA (cDNA) encoding the human wild-type mitochondrial ND4 protein. It is believed to restore the functional ND4 protein, thereby preventing the neuronal degeneration of retinal ganglion cells, as demonstrated in a rat model of LHON (11). In a phase 1/2 study conducted in 15 LHON patients harboring the m.11778G>A *ND4* mutation (hereafter named *MT-ND4* patients), a single intravitreal injection (IVT) of lenadogene nolparvovec was well-tolerated and associated with a clinically significant improvement in visual function outcomes (12, 13). A visual benefit induced by lenadogene nolparvovec was recently suggested by two randomized, double-masked, sham-controlled phase three studies REVERSE [patients with a vision loss between 6 and 12 months; NCT02652780 (14)] and RESCUE [patients with a vision loss below 6 months; NCT02652767 (15)]. In both studies, lenadogene nolparvovec was injected in one eye, while the fellow eye received a sham injection, with the unexpected result of sustained visual improvement in both eyes. At 96 weeks post-injection, the mean gain from nadir (worst vision point) in REVERSE and RESCUE studies was, respectively, +28 and +26 in treated eyes and +24 and +23 ETDRS letters in sham eyes (14, 15). The REVERSE and RESCUE patients are currently followed in an extension study for up to 5 years after injection (CLIN06, NCT03406104). This bilateral improvement after the unilateral injection of a gene therapy product has also been observed in other clinical studies of LHON (16, 17). A mechanistic explanation could be transfer of the viral vector from the injected eye to the contralateral eye through the optic chiasm, as suggested by a non-human primate biodistribution study using unilaterally injected lenadogene nolparvovec (14).

In order to better characterize the efficacy of gene therapy in *MT-ND4* LHON patients, we indirectly compared the evolution of visual outcomes of treated patients in the REVERSE, RESCUE, and CLIN06 studies to the spontaneous evolution of natural history patients from a LHON registry study and previously published reports of LHON patients with visual outcome data used as an external control.

## Methods

### Patients Treated With Gene Therapy—Efficacy Pool

We analyzed the evolution of best corrected visual acuity (BCVA) from a pooled dataset of 76 LHON patients treated with a single IVT injection of lenadogene nolparvovec. The BCVA data were collected from study inclusion to week 96 after treatment in REVERSE (NCT02652780) (14) and RESCUE (NCT02652767) (15) and from week 96 after treatment to the last available observation in the ongoing long-term follow-up CLIN06 study of REVERSE and RESCUE (NCT03406104) (see Supplementary Table 1).

The study design and results of REVERSE and RESCUE have been previously reported (14, 15). Briefly, REVERSE and RESCUE were randomized, double-masked, sham-controlled, multi-center phase 3 clinical trials with a similar design, aiming at evaluating the efficacy and safety of lenadogene nolparvovec in LHON patients. The right eye of each subject was randomly allocated to receive either lenadogene nolparvovec or sham treatment in a 1:1 allocation ratio. The fellow (left) eye received the treatment not allocated to the right eye. Lenadogene nolparvovec at 9 × 10^10^ viral genomes (vg)/eye was administered once *via* a single IVT. Sham IVT injection was performed once by applying pressure to the eye at the location of a typical procedure using the blunt end of a syringe without a needle. Both studies enrolled symptomatic LHON patients aged 15 years or older and harboring the m.11778G>A *ND4* mutation. The only difference between the two studies was the timing of the onset of vision loss: from 181 to 365 days in both eyes in REVERSE and ≤ 180 days in the first-affected eye in RESCUE. A total of 37 patients (REVERSE) and 39 patients (RESCUE) were enrolled and treated.

All REVERSE and RESCUE patients who completed the study up to week 96 after injection were offered to participate in the extension CLIN06 study, for a total of 5 years of follow-up after injection. A total of 62 patients (31 from REVERSE and 31 from RESCUE) were enrolled in the extension study which is ongoing. For our analysis, we used all available BCVA data at the time of this report, including assessments up to 4 years after injection. Based on clinical results and non-human primate data (14), both treated and sham eyes were considered exposed to the study drug and pooled in the treated patient group.

The protocols of all three studies (RESCUE, REVERSE, and CLIN06) were approved by local independent ethics committees, and informed consent was obtained from all participants. All studies were performed in compliance with Good Clinical Practice and adhered to the ethical principles outlined in the Declaration of Helsinki.

### Natural History Patients—External Control Group—Natural History Pool

Natural history patients (those not treated with lenadogene nolparvovec, although they could have been treated with idebenone), with age and LHON genotype adjusted to those of treated patients, were used as an external control for the analysis. To this end, we created a large database containing visual outcome data from 11 studies originating from two main sources: (i) the REALITY LHON registry (NCT03295071) (18) sponsored by GenSight Biologics and (ii) 10 published studies on LHON identified after a systematic review of the literature (3, 19–27). Studies were included in the database only if they reported individual (patient- and eye-level) visual acuity values along with documentation of the time after vision loss in cohorts of at least five *MT-ND4* patients. For relevant comparison with treated patients, we included only patients from the pooled database who matched the inclusion criteria of REVERSE and RESCUE as regards age and LHON genotype (i.e., symptomatic LHON patients carrying the m.11778G>A *ND4* mutation who were 15 years or older at the onset of vision loss). Further details on REALITY and on the systematic literature review are provided in the Supplementary Methods and Supplementary Table 1.

### Handling of Data

For the analyses, all visual acuity values (from treated and natural history patients) were converted to logarithm of the minimal angle of resolution (LogMAR) using standard formula for on-chart eyes (28) and the following conventions for off-chart eyes: patients only able to count fingers or detect hand motion were assigned LogMAR values of +2.0 and +2.3, respectively, according to the Lange scale (29); light perception and no light perception visual acuities were assigned LogMAR values of +4.0 and +4.5, respectively, to align with the equivalence used in the lenadogene nolparvovec studies and the REALITY registry (14, 15, 18). All eyes were assigned a LogMAR value of 0 at 1 month before the onset of vision loss, in line with the normal visual acuity of LHON mutation carriers before expression of the disease as described in the literature (30, 31) and pre-symptomatic data of lenadogene nolparvovec studies and REALITY registry. All extracted data and conversions of visual acuity values to LogMAR underwent a thorough quality control process for ensuring the accuracy of all LogMAR reported values.

### Statistical Methods

All data from treated and natural history patients were imported in a pooled database for the analyses. All analyses were performed at the patient level and at the eye level.

In a first step, we explored graphically the evolution of visual acuity in treated and natural history eyes more than 12 months after vision loss, when all REVERSE and RESCUE patients would have been treated with rAAV2/2-*ND4*, using a locally estimated scatterplot smoothing (LOESS), non-parametric, local regression model in which each patient's eyes were considered independently. Smoothing parameters were based on the corrected Akaike Information Criterion (SAS default method with values from 0.3 to 0.6). LOESS curves with 95% confidence interval (CI) were presented from 12 months up to 52 months after vision loss, corresponding to the maximal duration of follow-up for treated eyes in the extension study. All subsequent visual acuity values of natural history eyes were assigned to the 52-month timepoint using the next observation carried backward method. Using this method, all visual acuity values from the efficacy and natural history pools could be plotted on the same figure.

In a second step, we compared the visual outcomes between treated eyes and natural history eyes at 12, 18, 24, 36, and 48 months after vision loss (when all treated eyes were on treatment) and at the last available visual acuity value. For the 12- to 48-month analysis, only the closest value to the nominal timepoint was selected for each eye based on pre-specified time windows (month 12: [9;15] months; Month 18: [15;21] months; Month 24: [21;30] months; Month 36: [30;42] months; Month 48: [42;54] months). Conversely, for the analysis at the last available visual acuity value, final visual acuity values from all eyes were considered in the analysis, maximizing the sample size. The following visual outcomes were analyzed: visual acuity values in LogMAR, eye response rates at a threshold of LogMAR ≤ 1.6 (on-chart values on the ETDRS scale) and LogMAR ≤ 1.3 (cutoff for blindness according to WHO), and eye response rates with an improvement from nadir ≥ 0.3. For improvement from nadir, only eyes with at least two visual acuity assessments were selected for the analyses. Comparisons of visual outcomes were performed by a non-parametric test (Kruskal–Wallis for visual acuity values and chi-square test for eye response rates). In addition, a parametric model with repeated measures on patients was also used for the analyses on both eyes in order to take into account the inter-eye correlation of each patient (mixed-model analysis of covariance for visual acuity values and generalized linear mixed model for eye response rate).

In order to control the potential confounding covariates in the comparative analysis, the treatment effect at last available visual acuity value was also estimated by a multivariate analysis with repeated measures on patients. Age at onset of vision loss, gender, and duration of follow-up were explored as covariates in the multivariate analysis.

Additional analyses were also performed separately considering only the better eye and worse eye of each patient. Better eye and worse eye were selected based on their visual acuity value at last available evaluation or at their nadir in cases of identical values at last available evaluation or on their mean value in cases of identical nadir values. For patients who had visual acuity data in one eye only, the eye was included in both better-eye and worse-eye analyses.

All statistical analyses were carried out with SAS® software version 9.4. Statistical significance was set at *P* < 0.05.

## Results

### Characteristics of the Analyzed Population

Among the 44 patients enrolled in the REALITY study, 23 were *MT-ND4* LHON aged 15 years or older. Among the 304 *MT-ND4* patients in the natural history studies, 185 met the inclusion criteria of age at onset ≥ 15 years and at least one available visual acuity value with time from onset of vision loss. Thus, a total of 208 eligible natural history patients (408 eyes) were used as the external control cohort for comparison with the 76 treated patients (152 eyes) from the REVERSE and RESCUE studies (see Supplementary Table 1 and Table 1 for details).

**Table 1:** Description of the population.

	**Treated (*N* = 76)**	**Natural history** (***N* = 208)**	**Total (*N* = 284)**	***P*-value**
Number of eyes with visual acuity values	152	408^a^	560	
**Gender**	
Male (%)	61 (80.3%)	142 (82.6%)	203 (81.9%)	0.67 (C)
Missing data	0	36^b^	36	
**Age at onset of vision loss (years)**	
Median	32.5	23.5	25.0	<0.01 (KW)
Range	15.0–69.0	15.0–71.0	15.0–71.0	
**Number of visual acuity assessments per patient**	
Median	26.0	2.0	2.0	<0.01 (KW)
Mean (SD)	26.8 (5.2)	4.1 (4.0)	10.2 (11.0)	
**Patient follow-up since vision loss (months)**^c^	
Median	39.8	25.3	34.6	0.01 (KW)
Range	8.1–51.5	0.0–768.0	0.0–768.0	
Q1–Q3	32.1–44.1	4.0–108.4	7.6–49.5	
Patients with follow-up >36 months	64.5%	38.0%	45.1%	<0.01 (C)
**Time from vision loss to treatment (months)**	
Median	6.5	NA	NA	NA
Range	2.3–12.8	NA	NA	

*C, chi-square test; KW, Kruskal–Wallis test; NA, not applicable; SD, standard deviation*.

a *Eight natural history patient had visual acuity values in one eye only, leading to a sample size of 408 eyes*.

b *All gender missing data were from the natural history study of Lam in 2014 (20)*.

c *Defined as the time from vision loss to the last available visual acuity value, regardless of the eye*.

The characteristics of the patients at onset of vision loss are described in Table 1. Overall, both treated and natural history patients were typical of the *MT-ND4* LHON population with a high proportion of males (81.9%) and a young age at onset of vision loss (median, 25 years). Natural history patients had a younger age of onset (median, 23.5 years) compared to treated patients (median, 32.5 years).

The mean number of visual acuity assessments per patient was larger in the treated group (26.8) as compared with the natural history group (4.1). Treated patients had a longer median follow-up duration after vision loss (39.8 months) than natural history patients (25.3 months). Conversely, the follow-up values of treated patients were distributed over a narrow range (25% of patients have been followed for more than 44.1 months; maximal follow-up, 51.5 months) as opposed to a wider distribution for natural history patients (25% of patients were followed for more than 108.4 months; maximal follow-up, 768 months).

The treated patients received lenadogene nolparvovec injection between 2.3 and 12.8 months after vision loss (median, 6.5 months). Half of the eyes (54%) had received treatment at month 6 ([3, 9] months) after vision loss, nearly all eyes (93%) at month 12 ([9, 15] months) after vision loss, and all patients (100%) at month 18 ([15, 21] months) after vision loss. We started the indirect comparison at month 12, which coincided with the time when nearly all eyes had received treatment.

### Global Evolution of Visual Acuity Over Time

**Figure 1:**
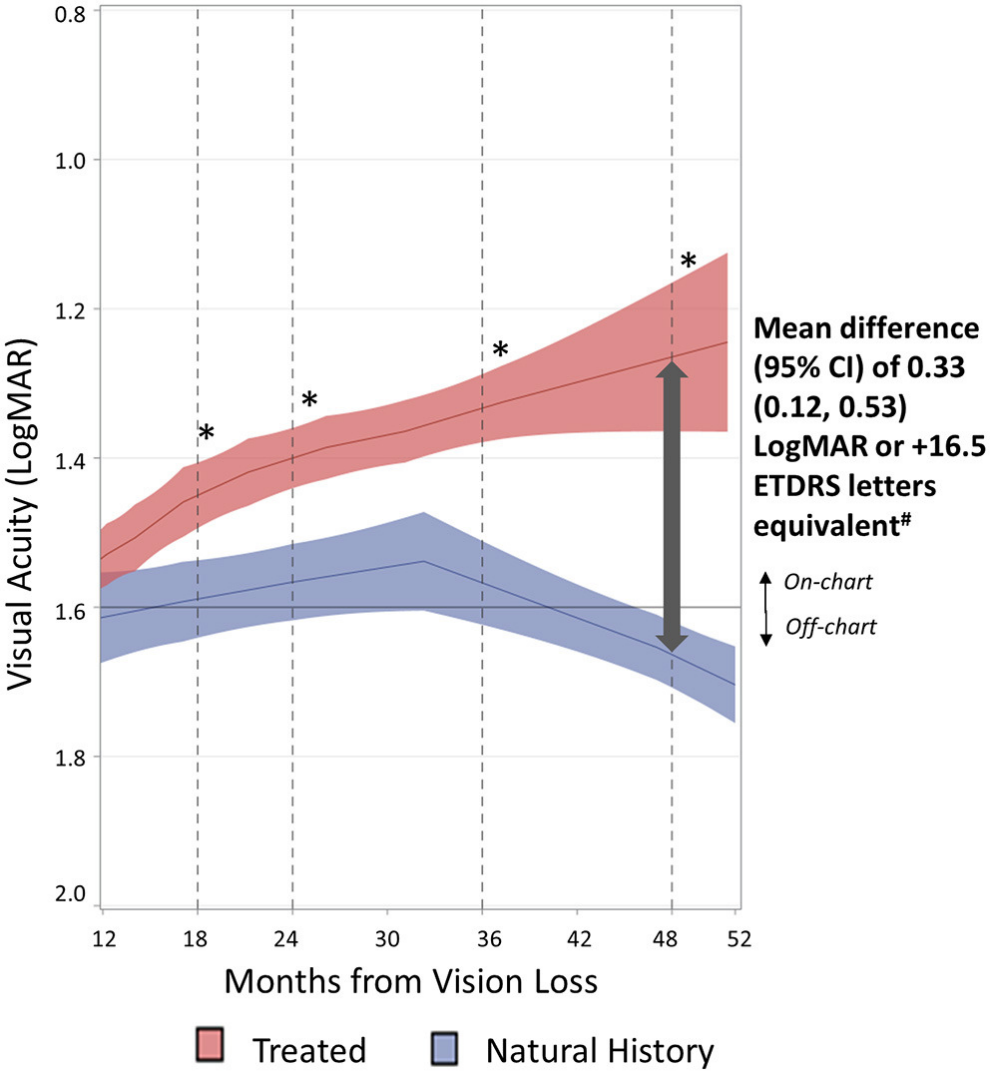
Evolution of visual acuities of treated eyes vs. natural history eyes. The evolution of visual acuities over time for treated eyes (*n* = 152) and natural history eyes (*n* = 408) was estimated by LOESS regression (solid line) with 95% confidence interval around the fitted curve (shaded area). Smoothing parameter: 0.332 for treated eyes and 0.408 for natural history eyes. *A statistically significant difference between treated and natural history eyes is illustrated by the non-overlapping confidence intervals (CI) of LOESS curves. ^**#**^Mean differences and 95% CI at month 48 were computed based on a separate analysis described in Table 2.

The LOESS regression curve for treated patients (in red in Figure 1; see Supplementary Figure 1 for the scatterplot) showed a progressive and sustained improvement of BCVA from month 12 up to month 52. Notably, the lowest point of the curve (corresponding to the worst visual acuities) remained on-chart, with BCVA values not exceeding 1.6 LogMAR.

The natural history patients showed a clear distinctive pattern from treated patients as illustrated by the LOESS regression curves shown in blue in Figure 1 (see Supplementary Figure 1 for the scatterplot). In natural history patients, visual acuities plateaued around 1.6 LogMAR, with no recovery over 2 years (up to month 36 after vision loss). A progressive and continuous decline to off-chart values was then noted from month 36 up to month 52. The 52-month timepoint shown in Figure 1 also takes into account all subsequent visual acuity values of natural history patients.

The divergence observed between the two curves from month 12 after vision loss is further evidenced by the absence of overlap in 95% CI at all later timepoints. Indeed the natural history patients showed an absence of visual recovery over the entire period from month 12 to 52, whereas the treated patients showed a gradual and consistent improvement over the same time period.

The treatment effect was also observed when REVERSE and RESCUE studies were analyzed separately, showing a similar improvement in the visual acuity LOESS curve of the treated patients vs. the external control group (see Supplementary Figures 2, 3).

Additionally, the treatment effect for both eyes was also observed when the better eye and worse eye of patients were analyzed separately.

### Comparison of Visual Acuities at Each Timepoint

Table 2 presents the results from month 12 to 48 after vision loss when treated eyes had been injected with lenadogene nolparvovec. In agreement with LOESS regression curves, quantitative analyses showed better visual acuity of treated eyes when compared with natural history eyes at all evaluated timepoints from month 12 to 48. The difference in mean visual acuity between treated and natural history eyes was statistically significant at all time points based on a non-parametric Kruskal–Wallis test and at month 36 and 48 based on a mixed ANCOVA model, taking into account the inter-eye correlation for each patient. The mean visual acuities with 95% CI for treated eyes and natural history eyes were, respectively, 1.26 (1.14, 1.37) and 1.59 (1.41, 1.76) LogMAR at month 48 (*p* < 0.01 for both Kruskal–Wallis and mixed ANCOVA), with a mean difference of 0.33 (0.12, 0.53) LogMAR in favor of treated eyes (Table 2). The median visual acuities for treated eyes and natural history eyes were, respectively, 1.30 vs. 1.62 LogMAR at month 48.

**Table 2:** Visual acuity of treated eyes vs. natural history eyes with time intervals from vision loss.

**Time from vision loss**	**Treated (*N* = 152 eyes)**	**Natural history** (***N* = 408 eyes)**
**Month 12—[9, 15] months**		
Number of eyes	150	76
Visual acuity (LogMAR)		
Median	1.55	1.70
Mean (SD)	1.57 (0.55)	1.69 (0.67)
95% CI (mean)	(1.48, 1.66)	(1.54, 1.84)
Mean difference (95% CI)	0.118^#^ (−0.047, 0.282)	
**Month 18—[15, 21] months**		
Number of eyes	149	57
Visual acuity (LogMAR)		
Median	1.40	1.60
Mean (SD)	1.46 (0.51)	1.60 (0.54)
95% CI (mean)	(1.38, 1.54)	(1.46, 1.75)
Mean difference (95% CI)	0.144^#^ (−0.017, 0.304)	
**Month 24—[21, 30] months**		
Number of eyes	146	80
Visual acuity (LogMAR)		
Median	1.40	1.52
Mean (SD)	1.40 (0.59)	1.54 (0.52)
95% CI (mean)	(1.30, 1.50)	(1.42, 1.65)
Mean difference (95% CI)	0.139^##^ (−0.016, 0.293)	
**Month 36—[30, 42] months**		
Number of eyes	128	66
Visual acuity (LogMAR)		
Median	1.30	1.55
Mean (SD)	1.33 (0.59)	1.52 (0.47)
95% CI (mean)	(1.23, 1.44)	(1.40, 1.63)
Mean difference (95% CI)	0.183^##^, ^*^ (0.018, 0.348)	
**Month 48—[42, 54] months**		
Number of eyes	62	27
Visual acuity (LogMAR)		
Median	1.30	1.62
Mean (SD)	1.26 (0.45)	1.59 (0.44)
95% CI (mean)	(1.14, 1.37)	(1.41, 1.76)
Mean difference (95% CI)	0.329^##^, ^**^ (0.125, 0.534)	

*CI, confidence interval; LogMAR, logarithm of the minimal angle of resolution; SD, standard deviation*.

*The time from vison loss was calculated for each eye of each patient. For each eye, only the closest value to the nominal timepoint was selected based on the time windows indicated in brackets*.

# *P < 0.05,*

## *P < 0.01: statistically significant differences vs. natural history eyes using Kruskal–Wallis test*.

* *P < 0.05,*

** *P < 0.01: statistically significant difference vs. natural history eyes using mixed ANCOVA with repeated measures on a patient*.

The treatment effect was also observed when REVERSE and RESCUE studies were analyzed separately vs. the external control group. The mean visual acuity difference with 95% CI between treated eyes and natural history eyes at month 48 was 0.31 (0.10, 0.52) LogMAR for REVERSE (*p* < 0.01 for both statistical tests) and 0.49 (0.08, 0.89) LogMAR for RESCUE (*p* = 0.03 for both statistical tests) in favor of treated eyes (see Supplementary Table 2).

Similar results were obtained when better eyes and worse eyes were analyzed separately, although a statistical significance was not reached at all timepoints (see Supplementary Table 3).

### Comparison of Visual Acuities at Last Available Observation

Table 3 presents the analyses at last available visual acuities comparing treated eyes vs. natural history eyes, with separate analyses performed for each study (REVERSE and RESCUE) and for better and worse eyes.

**Table 3:** Visual acuity of treated eyes vs. natural history (NH) eyes at last observation.

	**Both eyes**				**Better eye**		**Worse eye**	
	**All treated**	**REVERSE**	**RESCUE**	**NH**	**All treated**	**NH**	**All treated**	**NH**
Number of eyes	152	74	78	408	76	208	76	208
Visual acuity (LogMAR)								
Median	1.40	1.30	1.40	1.70	1.30	1.60	1.45	2.00
Mean (SD)	1.36 (0.60)	1.29 (0.47)	1.43 (0.71)	1.68 (0.61)	1.24 (0.54)	1.57 (0.62)	1.48 (0.64)	1.79 (0.57)
95% CI (mean)	(1.27, 1.46)	(1.18, 1.40)	(1.27, 1.59)	(1.62, 1.74)	(1.12, 1.37)	(1.49, 1.65)	(1.33, 1.63)	(1.71, 1.86)
Mean difference vs. NH (95% CI)	0.31^##^, ^**^ (0.20, 0.43)	0.38^##^, ^**^ (0.24, 0.53)	0.25^##^, ^**^ (0.10, 0.40)	-	0.32^##^ (0.17, 0.48)	-	0.31^##^ (0.15, 0.46)	-

*CI, confidence interval; NH, natural history; LogMAR, logarithm of the minimal angle of resolution; SD, standard deviation*.

## *P < 0.01: statistically significant differences vs. NH eyes using Kruskal–Wallis test*.

** *P < 0.01: statistically significant difference vs. NH eyes using mixed ANCOVA with repeated measures on a patient*.

The mean visual acuities with 95% CI for treated and natural history eyes were, respectively, 1.36 (1.27, 1.46) and 1.68 (1.62, 1.74) LogMAR at the last observation (*p* < 0.01 for both Kruskal–Wallis and mixed ANCOVA), with a mean difference of 0.31 (0.20, 0.43) LogMAR in favor of treated eyes. The median visual acuities for treated eyes and natural history eyes were, respectively, 1.40 and 1.70 LogMAR.

For REVERSE and RESCUE studies, the mean BCVAs with 95% CI for treated eyes were 1.29 (1.18, 1.40) and 1.43 (1.27, 1.59) LogMAR, respectively, while the mean visual acuity for natural history eyes was 1.68 (1.62, 1.74) LogMAR. The mean difference with 95% CI between REVERSE treated eyes and natural history eyes was 0.38 (0.24, 0.53) LogMAR in favor of treated eyes (*p* < 0.01 for both statistical tests). The mean difference between RESCUE treated eyes and natural history eyes was 0.25 (0.10, 0.40) LogMAR in favor of treated eyes (*p* < 0.01 for both statistical tests). The median visual acuity was 1.30 for REVERSE and 1.40 for RESCUE, vs. a median LogMAR of 1.70 for natural history eyes.

For analyses performed on better eyes, the mean visual acuities with 95% CI for treated and natural history eyes were, respectively, 1.24 (1.12, 1.37) and 1.57 (1.49, 1.65) LogMAR at the last observation (*p* < 0.01 for Kruskal–Wallis test), with a mean difference of 0.32 (0.17, 0.48) LogMAR in favor of treated eyes. For analyses performed on worse eyes, the mean visual acuities with 95% CI for treated eyes and natural history eyes were, respectively, 1.48 (1.33, 1.63) and 1.79 (1.71, 1.86) LogMAR at the last observation (*p* < 0.01 for Kruskal–Wallis test), with a mean difference of 0.31 (0.15, 0.46) LogMAR in favor of treated eyes.

### Multivariate Analysis of Visual Acuities

We performed a multivariate analysis to explore the potential impact of age at onset, gender, and duration of follow-up on the treatment effect at last visual acuity observation. Both age at onset (*p* = 0.0050) and follow-up duration (*p* = 0.0108) showed a statistically significant effect on visual acuity: younger patients and those with a shorter follow-up had better visual acuity independent of treatment. In contrast, gender had no effect on visual acuity outcome (*p* = 0.9236).

When considering the significant covariates in the analysis (age at onset and duration of follow-up), the treatment effect was confirmed in favor of treated eyes, with a statistically significant least squares mean difference in visual acuity of 0.32 (0.20, 0.44) LogMAR as compared with natural history eyes (*p* < 0.0001) (Table 4).

**Table 4:** Visual acuity in treated eyes vs. natural history eyes at last observation—multivariate analysis with age and duration of follow-up as covariates.

	**Treated** (***N* = 152 eyes)**	**Natural history (*N* = 408 eyes)**
Number of eyes	152	408
Time from vision loss to last observation (months)	
Median (range)	39.9 (8.1–51.5)	28.4 (0.0–768.0)
Visual acuity (LogMAR)	
LS means (95% CI)	1.36 (1.26, 1.46)	1.68 (1.62, 1.74)
Effect estimate with 95% CI	0.32 (0.20, 0.44)^***^	

*CI, confidence interval; LogMAR, logarithm of the minimal angle of resolution; LS, least squares*.

*** *P < 0.0001: statistically significant treatment effect using multivariate analysis, with age and duration of follow-up as covariates (repeated measures on a patient)*.

### Eye Response Rates

**Figure 2:**
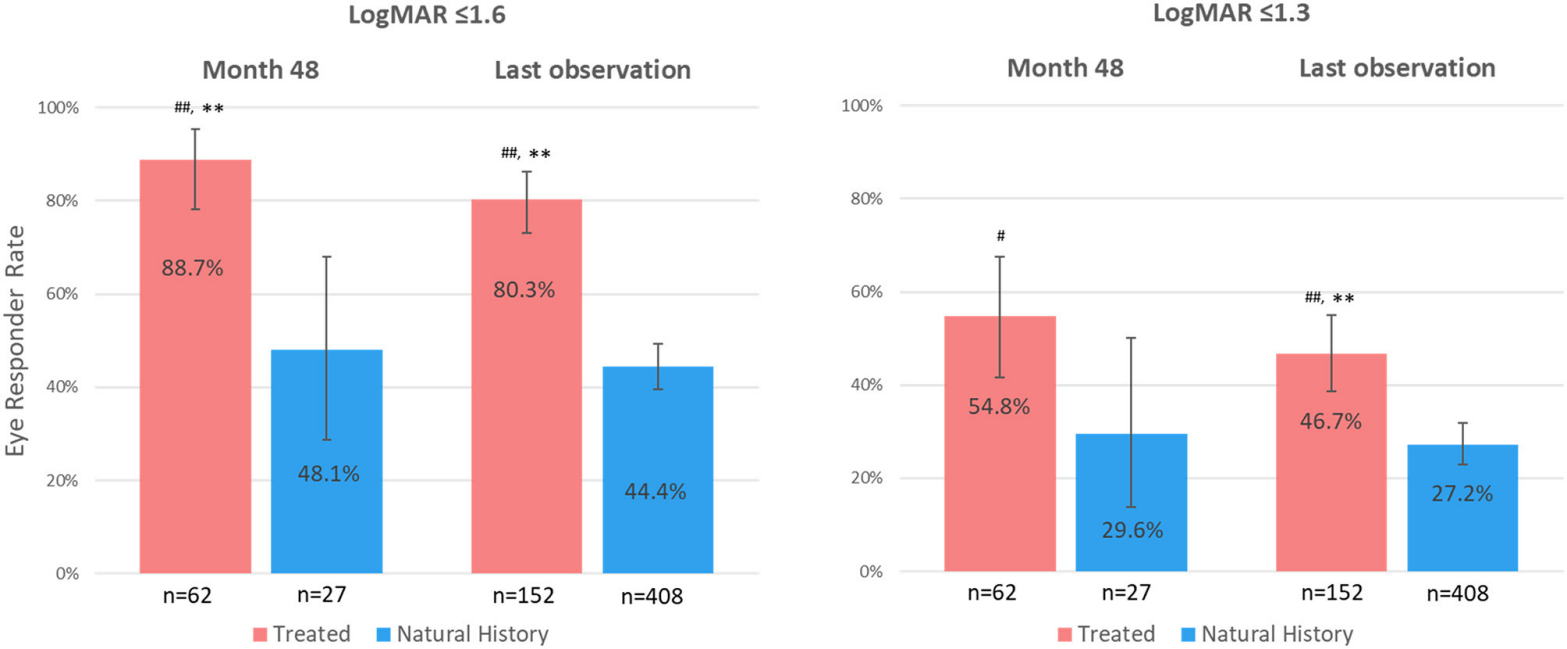
Eye responder rates at month 48 since vision loss and at last observation. LogMAR, logarithm of the minimal angle of resolution; *n*, number of eyes. Response rates (%) are defined as the proportion of eyes with visual acuity values ≤ 1.6 LogMAR (left panel) or ≤ 1.3 LogMAR (right panel). Error bars represent 95% confidence interval. ^#^*P* < 0.05, ^##^*P* < 0.01: statistically significant difference vs. natural history eyes using chi-square test. **P < 0.01: statistically significant difference vs. natural history eyes using a generalized linear mixed model with repeated measures on a patient.

At month 48 after vision loss, most (55/62) treated eyes [88.7%; 95% CI (78.1, 95.3)] were on-chart (LogMAR ≤ 1.6) as compared to less than half of the eyes (13/27) in the natural history group [48.1%, 95% CI (28.7, 68.1)] (Figure 2, left panel). The difference was statistically significant at *p* < 0.01 with both statistical tests (with or without considering the inter-eye correlation of each patient). Comparable results were observed for the response rates using the 1.3-LogMAR threshold (cutoff for blindness according to WHO criteria) (Figure 2, right panel). At month 48, 34/62 treated eyes [54.8%; 95% CI (41.7, 67.5%)] were responders as compared to 8/27 [29.6%, 95% CI (13.8, 50.2)] in the natural history group (*p* = 0.03 with chi-square test).

At last observation, 122/152 treated eyes [80.3%; 95% CI (73.0, 86.3)] were on-chart (LogMAR ≤ 1.6), as compared to 181/408 natural history eyes [44.4%; 95% CI (39.5, 49.3%)], with a statistically significant difference with both statistical tests (*p* < 0.01) (Figure 2, left panel). Similar results were observed when evaluating the REVERSE and RESCUE studies separately, with 85.1% [95% CI (75.0, 92.3)] and 75.6% [95% CI (64.6, 84.7)] of treated eyes being on-chart at last observation, respectively (*p* < 0.01 with both statistical tests vs. natural history eyes).

When using the 1.3-LogMAR response threshold at last observation, 71/152 treated eyes [46.7%; 95% CI (38.6, 55.0)] were responders as compared to 111/408 natural history eyes [27.2%; 95% CI (22.9, 31.8)] (*p* < 0.01 with both statistical tests) (Figure 2, right panel).

The proportion of eyes showing a meaningful improvement from nadir of at least 0.3 LogMAR was greater in the treated group as compared to the natural history group: 35/62 [56.5%; 95% CI (43.3, 69.0)] of treated eyes vs. 4/25 [16.0%; 95% CI (4.5, 36.1)] of natural history eyes at month 48 [*p* < 0.01 (chi-square) and *p* = 0.02 (repeated measures)] and 75/152 [49.3%, 95% CI (41.1, 57.6)] of treated eyes vs. 36/127 [28.3%; 95% CI (20.7, 37.0)] of natural history eyes at last observation (*p* < 0.01 with both statistical tests).

The treatment effect on responder rates observed for both eyes was similar to that seen when considering better eyes or worse eyes.

## Discussion

In this pooled analysis, we demonstrated a sustained and clinically relevant improvement in the visual acuities of *MT-ND4* LHON patients treated with lenadogene nolparvovec when compared to the spontaneous evolution of vision in an external control group comprised of natural history genetically and age-matched LHON patients.

The RESCUE and REVERSE studies demonstrated bilateral visual improvement after unilateral injection with lenadogene nolparvovec in *MT*-*ND4* LHON patients (14, 15). As the visual improvement was observed in both treated and sham eyes, the primary endpoint, which was based on the difference from baseline between sham and treated eyes, was not met in both studies. RESCUE included subjects at an earlier stage of LHON who had not yet reached their nadir at the time of treatment, so it is perhaps not unexpected that visual outcomes at week 96 had not recovered to the baseline values along the early curve of anticipated visual decline despite clear improvement from nadir. Indeed, from a clinical standpoint, the level of visual improvement achieved from nadir was substantial in both studies, with a mean gain in ETDRS letters at week 96 after treatment of +28 and +24 in REVERSE and of +26 and +23 in RESCUE for treated eyes and sham eyes, respectively. Surprisingly, the earlier treatment (within 6 months after vision loss) in RESCUE did not provide better outcomes than in REVERSE (between 6 and 12 months after vision loss). However, these results are still striking considering that a clinically relevant improvement of visual acuity remains an uncommon feature in *MT*-*ND4* LHON subjects.

In a recent meta-analysis of untreated *MT-ND4* LHON patients aged at least 15 years at onset of vision loss reported in the world literature, only 11.3% of patients showed some spontaneous visual recovery, although the definitions used for recovery varied among studies (10). In contrast, the great majority of treated patients (81% for REVERSE and 71% for RESCUE) had a clinically relevant recovery in BCVA from nadir at week 96 after treatment when using a valid recovery endpoint endorsed by an international group of experts (7, 15). While these results are strongly supportive of a treatment-related effect in REVERSE and RESCUE, the absence of an adequate placebo group of untreated subjects in both studies precludes drawing definitive conclusions regarding treatment benefit.

Here we try to address this issue by comparing the visual outcomes of treated patients to an external control group of individual natural history *MT-ND4* LHON patients. The use of an external control in supporting treatment efficacy is acknowledged by many regulatory agencies, and this approach has been increasingly used in rare diseases and in oncology trials where the use of a placebo group is not ethically feasible (32, 33). In this regard, a number of groups, including ours, have conducted LHON registry studies for use as external comparators in drug trials (20, 26, 34). However, these registries are usually limited in size owing to the rarity of the disease, thereby limiting the statistical power of such comparisons. In our own LHON registry study, REALITY, we included a total of 44 LHON patients, of whom only 23 met the inclusion criteria of REVERSE and RESCUE trials (18). In order to reach a sufficient sample size to enable a statistically meaningful comparison with treated patients, we complemented our natural history dataset with patient-level data identified through a systematic review of the literature. This allowed us to build a large database of 208 natural history LHON patients who shared the same characteristics as those included in REVERSE and RESCUE.

This work is the first to thoroughly describe the spontaneous evolution of visual acuity after vision loss in such a large cohort of natural history *MT-ND4* LHON patients. We showed that, in natural history eyes, after the initial acute phase of visual acuity decline to nadir, visual acuity plateaued with no recovery over 2 years (from month 12 to 36 after vision loss). These data are consistent with the known temporal course of clinical and pathophysiological changes in LHON described in the literature (2, 5, 35). Interestingly, in our study, the stable phase of natural history patients was followed by a trend for a later decline in visual acuity from 3 years after onset, suggesting that visual outcomes may continue to deteriorate.

The first thorough investigation of the natural history of visual function of LHON patients dates back to 1963 when van Senus provided a detailed individual description of a Dutch cohort of 27 LHON pedigrees, of whom 12 were later molecularly confirmed as carrying the *MT*-*ND4* mutation (36, 37). While we could not include these patients in our analyses due to the imprecision of reported visual acuity values (available only as ranges) and uncertainty regarding the timing of measurements after vision loss, these natural history data are overall coherent with those described in our analyses. Indeed the majority of MT-*ND4* patients aged 15 years or older from the van Senus cohort had a poor visual acuity at the time of investigation, with 110/121 (91%) eyes having visual acuity worse than 6/60 (20/200; LogMAR +1.0), 100/121 (83%) eyes worse than 3/60 (20/400; LogMAR +1.3), and 69/121 (57%) eyes worse than 6/300 (20/1,000; LogMAR +1.7). Among those *MT-ND4* patients for whom two visual acuity points in time were reported, only 5/54 (9%) eyes in four patients had documented improvement in visual acuity of at least −0.2 LogMAR between onset of vision loss and time of investigation (36).

In our analysis, a young age at onset was an independent predictor of better visual outcomes. A better prognosis is known to be driven by onset in children of 12 years old or younger (10, 38–40). Here we report that better visual outcomes at younger ages may hold true for patients aged 15 years or older at onset.

In contrast to the evolution seen in natural history eyes, treated eyes followed a clearly distinct pattern, with a sustained, continuous, and progressive improvement in visual acuity from 12 to 52 months after the onset of vision loss. At month 48, there was a statistically significant and clinically relevant difference in the mean visual acuity of 0.33 LogMAR (+16.5 ETDRS letters equivalent) in favor of treated eyes as compared to natural history eyes (*p* < 0.01). This level of improvement translated into a better quality of life for patients in both the REVERSE and RESCUE studies, especially for mental health, dependency, and role difficulty dimensions as measured by the National Eye Institute Visual Function Questionnaire-25 (14, 15). It is noteworthy that the improvement exceeded +15 ETDRS letters, a response level recognized as clinically relevant by regulators (41). Moreover, the magnitude of the treatment effect was not impacted when known confounding variables (onset age and duration of follow-up) were accounted for in multivariate analyses, with a statistically significant and clinically relevant mean difference of 0.32 LogMAR (+16 ETDRS letters) at the last visual acuity measurement in favor of treated eyes. As regards responder rates, most treated eyes (89%) were on-chart at month 48, compared to less than half (48%) of the natural history eyes (cutoff for response: LogMAR ≤ 1.6). When using the 1.3-LogMAR cutoff for blindness, the responder rates at month 48 (LogMAR ≤ 1.3) were 55% for treated eyes vs. 30% for natural history eyes. Similarly, a higher proportion of treated eyes had a gain from nadir of at least 0.3 LogMAR when compared with natural history eyes (57 vs. 16% at month 48). However, the nadir of natural history studies is not as well-documented as the nadir from RESCUE and REVERSE trials, hence limiting the interpretation of these findings.

Importantly, we included all treated and sham eyes of unilaterally injected patients in our analyses based on the assumption that sham eyes were exposed to lenadogene nolparvovec, presumably by transfer of the viral vector through the optic chiasm (14). Interestingly, the significant treatment effect vs. natural history eyes observed when considering all eyes was maintained in the analyses performed separately on better eyes and on worse eyes, as seen in Table 3. A study investigating the effect of unilateral vs. bilateral injection of lenadogene nolparvovec in *MT-ND4* LHON patients is underway and should provide more information on the potential additional benefit provided by bilateral treatment with lenadogene nolparvovec (NCT03293524).

We previously reported that lenadogene nolparvovec improved visual outcomes up to 96 weeks after treatment in REVERSE and RESCUE, which corresponds to ~2.5 years after vision loss (14, 15). Here we extend these findings by showing the persistence of visual benefit in the long term, with continuous improvement in BCVA up to the last available observation [i.e., 51.5 months (4.3 years) from vision loss]. Moreover, the long-term trend depicted in the regression analyses suggests that visual improvement in treated patients may continue to progress with time, in line with the improvement noted up to 7 years after treatment in a small cohort of *MT-ND4* LHON patients treated with another gene therapy product, although the majority of patients in that study were younger than 15 years in age (16). Further results of the ongoing CLIN06 study with follow-up planned for 5 years after treatment (~6 years after vision loss) should provide similar information for patients treated with lenadogene nolparvovec.

To enable a fair comparison in a non-randomized setting and even more so when using an external comparator, it is essential that treated and control groups share comparable characteristics. To ensure the comparability of groups, we carefully selected natural history patients who would have been eligible for our gene therapy trials as regards the age of onset and genotype, two criteria that have been shown to be major determinants of spontaneous recovery in LHON (10). In this report, both treated and natural history groups were typical of the general *MT-ND4* LHON population described in the literature, with a predominance of male patients (around 80%) and a median age at onset in the 20-30 s (5). It should be noted, however, that when compared with treated patients, natural history patients were relatively younger (median age of 23 years vs. 32 years at onset) and were followed for a shorter period of time (median follow-up of 25 vs. 40 months). However, because both a younger age at onset and a shorter follow-up (when visual acuity may not have yet reached its worst level) are associated with a better visual outcome, this imbalance between groups was more likely to disadvantage treated eyes, thus reinforcing the significance of the observed difference between groups. Indeed when these two confounding variables were considered in the multivariate analyses, the difference in visual acuities between treated and natural history eyes was statistically significant (0.32 LogMAR difference in favor of treated eyes, *p* < 0.0001). Furthermore, it should be emphasized that we also retained natural history patients who may have been treated with idebenone, further potentially disfavoring the treated patients who, by study exclusion criteria, were not taking this medication.

Our study has several limitations. Although we used a systematic approach in selecting natural history data from the literature, we excluded studies containing only aggregated patient visual acuity data, which could have led to a potential bias in the inclusion of certain patients. Despite this reduction of the natural history database related to methodological concerns to enable rigorous indirect comparison analyses, the external control group ultimately included a substantial number of patients representative of the *MT-ND4* LHON population. The LOESS method used for describing the evolution of visual acuity is a non-parametric approach which does not take into account the intra-patient correlation, leading to a possible under-estimation of the confidence interval around the fitted curves. As such, the LOESS analysis should be regarded as descriptive in nature rather than inferential. However, we also performed formal statistical tests that took into account the intra-patient correlation, hence supporting the generalizability of the treatment effect. Furthermore, the treatment effect observed for both eyes was similar to that seen for better and worse eyes, indicating that the impact of inter-eye correlation is minimal on treatment effect. While the time from vision loss to treatment for all 152 treated eyes was 12.8 months, we chose to present our results from month 12. Indeed this does not have any impact on the interpretations of findings, as nearly all eyes (93%) had received the treatment by month 12.

Another concern relates to the heterogeneity of the collected visual acuities in our natural history cohort. Crucially, very few of the natural history studies specified that the visual acuities obtained were BCVAs, and there was no standardized assessment of vision as in rigorously performed clinical trial studies where LogMAR vision is measured using the ETDRS chart using a set protocol. Furthermore, most of the natural history studies were retrospective reviews of their patient cohorts, with most natural history data being cross-sectional (recorded at individual points in time) as opposed to longitudinal for treated eyes (several measurements over time). This difference in the frequency and number of visual acuity assessments per patient may have had an impact on the precision of the visual acuity LOESS model curves for the natural history patients, more so if visual acuity was not always determined with the optimal refraction. However, we believe this had a limited impact on the modelized curves because of the observed stability of the visual acuities over time across the different natural history studies. Furthermore, the most important outcome for the patient is the final visual outcome (last available visual acuity) which was largely reported many months after visual loss onset in the natural history studies that we included in our analyses.

Ultimately, a randomized trial vs. a true parallel placebo group would be the ideal next step to enhance the efficacy of gene therapy with lenadogene nolparvovec. However, there are many barriers to this approach, both operationally and ethically. Among the former include the potentially confounding concurrent, but non-uniform, use of idebenone and the challenges of recruitment for this rare disease. Ethical concerns would include the safety of intravitreal injection of placebo and the absence or delay of treatment in this neurodegenerative disease that is rapidly non-reversible.

In summary, we demonstrate that gene therapy with lenadogene nolparvovec induced a progressive, sustained, and statistically significant improvement of visual acuity up to more than 4 years after vision loss in LHON patients carrying the m.11778G>A mutation when compared to the spontaneous evolution of a large group of matched natural history patients used as an external comparator. Similarly, the same analyses applied to better and worse eyes and to the REVERSE and RESCUE study populations, considered independently, show similar results. The sensitivity analyses controlling for potential confounding factors confirm the robustness of the indirect comparison results. Finally, the strong temporal relationship between the start of improvement and administration of treatment and the size of the treatment effect observed further support the validity of our findings.

## Data Availability Statement

The raw data supporting the conclusions of this article will be made available by the authors, without undue reservation.

## Ethics Statement

The studies involving human participants were reviewed and approved by For REALITY: Western Copernicus Group IRB (for all US sites); Wills Eye Hospital IRB; Emory University IRB; UCLA Office of Human Research Protection Program; Baylor IRB; Massachusetts Eye and Ear IRB; CPP Sud-Ouest & Outre-Mer 4 (for all French sites); Comitato Etico Area Vasta Emilia Centro; Comitato Etico IRCCS Ospedale San Raffaele di Milano; Agencia Española del Medicamento y Productos Sanitarios (for all Spanish sites); CEICm idcsalud a Catalunya; Health Research Authority (for all UK sites); NRES Committee London Bloomsbury (for all UK sites); Moorfields Eye Hospital NHS Foundation Trust For RESCUE/REVERSE: West London & GTAC Research Ethics Committee; Ethics Committee of the LMU Munich; Comitato Etico Interaziendale Bologna-Imola (CE-BI); CPP Ile-de-France V; Emory IRB; UCLA IRB; Wills Eye Hospital IRB For CLIN06: Wills Eye Hospital IRB; CPP Sud-Est III; LMU Ethikkommission bei der LMU Munchen; NHS South Central - Oxford A Research Ethics Committee; UCLA Institutional Biosafety Committee; Emory University IRB; Comitato Etico di Area Vasta Emilia.

## Author Contributions

NN, PY-W-M, VC, VB, CV-C, RS, CJ, JS, FM, MR, MT, and J-AS contributed to the conception and design of the studies. NN, PY-W-M, VC, VB, MM, CV-C, RS, TK, AS, J-FG, CL, AD, NJ, CP, and RK contributed to data collection. CJ and FM contributed to statistical analyses. NN, PY-W-M, VC, VB, MM, CV-C, RS, TK, AS, J-FG, CL, AD, NJ, CP, RK, CJ, JS, FM, MR, MT, and J-AS contributed to analysis and interpretation. JS, NN, PY-W-M, and MT contributed to writing and are major contributors in the revisions. All the authors contributed to manuscript revision and read and approved the submitted version.

## Conflict of Interest

NN is a consultant for GenSight, Santhera Pharmaceuticals, and Stealth BioTherapeutics, has received research support from GenSight and Santhera Pharmaceuticals, has served on the Data Safety Monitoring Board for the Quark NAION study, and is a medical–legal consultant. PY-W-M is a consultant for GenSight and Stealth BioTherapeutics and has received research support from GenSight and Santhera Pharmaceuticals. VC is a consultant for Santhera Pharmaceuticals, GenSight, and Stealth BioTherapeutics and has received research support from Santhera Pharmaceuticals and Stealth BioTherapeutics. VB is a consultant for GenSight, Santhera Pharmaceuticals, and Stealth BioTherapeutics and has received research support from GenSight and Santhera Pharmaceuticals. MM is a consultant for GenSight Biologics and has received research support from GenSight. CV-C is a consultant for GenSight Biologics and Santhera Pharmaceuticals. RS is a consultant for GenSight Biologics. TK is a consultant for Santhera Pharmaceuticals, Chiesi, and GenSight Biologics and has received research support from Santhera Pharmaceuticals, GenSight Biologics, and Stealth BioTherapeutics. AS is a consultant for Stealth BioTherapeutics. CJ is an employee of eXYSTAT and a consultant for GenSight Biologics. FM is a co-founder of eXYSTAT and a consultant for GenSight Biologics. JS is a consultant for GenSight Biologics. MR and MT are GenSight Biologics employees. J-AS is a co-founder and shareholder of GenSight Biologics and a patent co-author on allotopic transport. The remaining authors declare that the research was conducted in the absence of any commercial or financial relationships that could be construed as a potential conflict of interest. The reviewer GS declared a past co-authorship with two of the authors NN, VB the handling editor.
