# Supplementary material for: Intravitreal Gene Therapy vs. Natural History in Patients With Leber Hereditary Optic Neuropathy Carrying the m.11778G>A ND4 Mutation: Systematic Review and Indirect Comparison
Source: Front Neurol. 2021 May 24;12:662838. doi: 10.3389/fneur.2021.662838 (PMC8181419; doi:10.3389/fneur.2021.662838)
Supplement: Supplementary file 1 [file Data_Sheet_1.docx]

# Supplemental Methods

*Natural History Pool*

*REALITY LHON Registry*

The REALITY registry (NCT03295071) was an observational, multi-center, natural history (NH) study on LHON in which visual outcomes were collected retrospectively based on medical chart abstractions. Main inclusion criteria were adult and pediatric patients with a confirmed LHON mutation (not restricted to m.11778G>A in *ND4*) and who had at least two visual function assessments between 1 and 3 years since vision loss. Patients treated with idebenone (Raxone, Chiesi) could be included in the study. None of the patients had received treatment with lenadogene nolparvovec.

The study was approved by the local independent Ethics Committees and a written informed consent was obtained for all included patients. The study was conducted in compliance with Good Clinical Practice and adhered to the ethical principles outlined in the Declaration of Helsinki.

A total of 44 patients were enrolled in eleven centers across Europe and United-States. Of these 44 patients, 23 patients who had the m.11778G>A *ND4* mutation (hereafter named *MT*-ND4 patients) and who were 15 years or older at onset of vision loss were included in our analysis (see **Supplementary Table S1**).

*Systematic literature review – Natural History Database*

We performed a systematic literature review to identify NH studies including *MT-ND4* LHON patients by searching the following databases: PubMed, Cochrane Review Library and Orpha.net. The search was performed in May 2020 and included the following keywords: “Leber hereditary optic neuropathy”, “LHON”, “ND4”, “G11778A”, “visual acuity”, “nadir”, “natural history”, “registry”, and “pedigree”. We limited our search to articles or abstracts published in English within a 30-year limit. Studies were included if they contained cohorts of at least 5 LHON patients with confirmed m.11778G>A mutation reporting individual (patient-level) visual acuity data. There were no restrictions on study designs, and both retrospective and prospective studies, registries or data collections were selected. Publications focusing on individual case reports were excluded because such reports are generally biased towards patients showing unusual disease clinical form. Studies where patients were treated with idebenone were retained, consistent with the inclusion criteria of REALITY.

In total, the systematic review yielded 19 literature studies (786 *MT-ND4* patients) out of which 10 studies (304 *MT-ND4* patients) provided patient-level information for cohorts ≥5 *MT-ND4* patients. Demographics and visual acuity data were extracted for the 304 patients across the 10 NH studies. All data extractions were initially performed by one reviewer in an Excel spreadsheet and subsequently verified against the source article by two independent reviewers for quality control. Data from natural history patients were imported in a pooled SAS database including the 304 *MT-ND4* patients with the following key variables: study ID, patient number, demographics (age at onset of vision loss and gender), and visual acuity data (value, eye assessed [left/right] and time of measurement since vision loss). Of the 304 *MT-ND4* patients, 185 fulfilled criteria for inclusion: confirmed age at onset ≥15 and at least one available visual acuity value with time from onset of vision loss.

*Natural History Pool – External Control Group*

The natural history pool included a total of 208 patients (23 from REALITY and 185 from the literature studies) who were carrying the m.11778G>A *ND4* mutation and were aged 15 years or older, and was used as the external control group for our indirect comparison analysis versus treated patients.
