# Supplementary material for: Intravitreal Gene Therapy vs. Natural History in Patients With Leber Hereditary Optic Neuropathy Carrying the m.11778G>A ND4 Mutation: Systematic Review and Indirect Comparison
Source: Front Neurol. 2021 May 24;12:662838. doi: 10.3389/fneur.2021.662838 (PMC8181419; doi:10.3389/fneur.2021.662838)
Supplement: Supplementary file 2 [file Data_Sheet_2.docx]

# Supplemental Material LHON Study Group

*Moorfields Eye Hospital, London, UK and UCL Institute of Ophthalmology, University College London, London, UK:* Patrick Yu-Wai-Man MD, PhD (Principal Investigator REVERSE, RESCUE, CLIN 06, International Principal Investigator REVERSE), James Acheson MD (Sub-Investigator REVERSE, RESCUE), Hayley Boston (REVERSE, RESCUE, CLIN 06), Maria Eleftheriadou MD (Sub-Investigator REVERSE, RESCUE), Simona Esposti MD (Sub-Investigator REVERSE, RESCUE, CLIN 06), Maria Gemenetzi (REVERSE, RESCUE), Lauren Leitch-Devlin (REVERSE, RESCUE), William R Tucker MD (Sub-Investigator REVERSE, RESCUE), Neringa Jurkute MD (Sub-Investigator REVERSE, RESCUE, CLIN 06); Asma Burale (CLIN 06)

*Emory University School of Medicine, Atlanta, Georgia, USA:* Nancy J. Newman MD (Principal Investigator REVERSE, RESCUE, CLIN 06, International Principal Investigator RESCUE, CLIN 06), Valérie Biousse MD (Sub-Investigator REVERSE, RESCUE, CLIN 06), G. Baker Hubbard MD (Sub-Investigator REVERSE, RESCUE), Andrew Hendrick MD (Sub-Investigator REVERSE, RESCUE), Michael Dattilo MD, PhD (Sub-Investigator REVERSE, RESCUE, CLIN 06), Jason Peragallo MD (Sub-Investigator REVERSE, RESCUE), Eman Hawy MD (Sub-Investigator REVERSE, RESCUE), Lindreth DuBois Med, MMSc, COMT (Study Coordinator REVERSE, RESCUE, CLIN 06), Deborah Gibbs COMT, CCRC, CCRP (Study Coordinator REVERSE, RESCUE, CLIN 06), Alcides Fernandes Filho MD (Study Coordinator REVERSE, RESCUE, CLIN 06), Jannah Dobbs (OCT/Photographer REVERSE, RESCUE, CLIN 06)

*IRCCS Istituto delle Scienze Neurologiche di Bologna, UOC Clinica Neurologica, Bologna, Italy, and Unit of Neurology, Department of Biomedical and Neuromotor Sciences (DIBINEM), University of Bologna, Bologna, Italy:* Valerio Carelli MD, PhD (Principal Investigator REVERSE, RESCUE, CLIN 06), Piero Barboni MD (Surgeon, IVT injections, REVERSE, RESCUE), Michele Carbonelli MD (Sub-Investigator REVERSE, RESCUE, CLIN 06), Lidia Di Vito MD (Sub-Investigator REVERSE, RESCUE), Giulia Amore MD (Sub-Investigator CLIN 06), Manuela Contin M.Sc (Pharmacist REVERSE, RESCUE), Susan Mohamed M.Sc (Pharmacist REVERSE, RESCUE), Chiara La Morgia MD, PhD (Sub-Investigator REVERSE, RESCUE, CLIN 06), Sara Silvestri (Technician REVERSE, RESCUE)

*Wills Eye Hospital and Sidney Kimmel Medical College of Thomas Jefferson University, Philadelphia, PA, USA:* Mark L. Moster MD (Principal Investigator REVERSE, RESCUE, CLIN 06), Robert C. Sergott MD (Head of the Central Reading Center, Annesley EyeBrain Center [AEBC], Vickie and Jack Farber Institute for Neuroscience at Jefferson Health Partnered with Wills Eye Hospital, for REVERSE, RESCUE, CLIN 06 studies), Melissa SantaMaria (Associate Director - Central Reading Center AEBC), Heather Tollis (Clinical Study Manager - Central Reading Center AEBC), Adam A. DeBusk MD (Sub-Investigator REVERSE, RESCUE, CLIN 06), Julia A. Haller MD (Surgeon, IVT injections, REVERSE, RESCUE); Maria Massini COT (Study Coordinator REVERSE, RESCUE, CLIN 06)

*Centre Hospitalier National d’Ophtalmologie des Quinze Vingts, Paris, France and Department of Neuro Ophthalmology and Emergencies, Rothschild Foundation Hospital, Paris, France:* José A. Sahel MD, PhD, Catherine Vignal MD (Principal Investigator CLIN 01, REVERSE, RESCUE, CLIN 06), Jean François Girmens MD (Surgeon, IVT injections, CLIN 01, REVERSE, RESCUE), Rabih Hage MD (Sub-Investigator CLIN 01, REVERSE, RESCUE, CLIN 06)

*Doheny Eye Institute / UCLA School of Medicine Los Angeles, CA, USA:* Alfredo A. Sadun MD, PhD (Principal Investigator REVERSE, RESCUE, CLIN 06), Gad Heilweil, Rustum Karanjia MD, PhD (Sub-Investigator), Irena Tsui

*Department of Neurology Friedrich-Baur-Institute, and Department of Ophthalmology, University Hospital, Ludwig-Maximilians-University Munich, 80336 Munich, Germany:* Thomas Klopstock MD (Principal Investigator, REVERSE, RESCUE, CLIN 06), Claudia B Catarino MD Sub-Investigator, REVERSE, RESCUE, CLIN 06), Claudia Priglinger MD (Sub-Investigator, REVERSE, RESCUE, CLIN 06), Siegfried Priglinger MD (Sub-Investigator, REVERSE, RESCUE, CLIN 06), Günther Rudolph MD (Sub-Investigator, REVERSE, RESCUE, CLIN 06), Stephan Thurau MD (Sub-Investigator, REVERSE, RESCUE, CLIN 06), Bettina von Livonius MD (Sub-Investigator, REVERSE, RESCUE, CLIN 06), Daniel Muth MD (Sub-Investigator, REVERSE, RESCUE, CLIN 06), Armin Wolf MD (Sub-Investigator, Surgeon, IVT injections, REVERSE, RESCUE), Jasmina Al-Tamami (Study Coordinator, REVERSE, RESCUE, CLIN 06), Angelika Pressler (Study Coordinator, REVERSE, RESCUE, CLIN 06), Cosima Schertler (Study Nurse, REVERSE, RESCUE, CLIN 06)

*TUMCells Interdisciplinary Center for Cellular Therapies, TUM School of Medicine, Munich, Germany:* Martin Hildebrandt MD (Sub-Investigator, REVERSE, RESCUE), Michael Neuenhahn, MD (Sub-Investigator, REVERSE, RESCUE)
