# Supplementary figures and images for: Intravitreal Gene Therapy vs. Natural History in Patients With Leber Hereditary Optic Neuropathy Carrying the m.11778G>A ND4 Mutation: Systematic Review and Indirect Comparison
Source: Front Neurol. 2021 May 24;12:662838. doi: 10.3389/fneur.2021.662838 (PMC8181419; doi:10.3389/fneur.2021.662838)

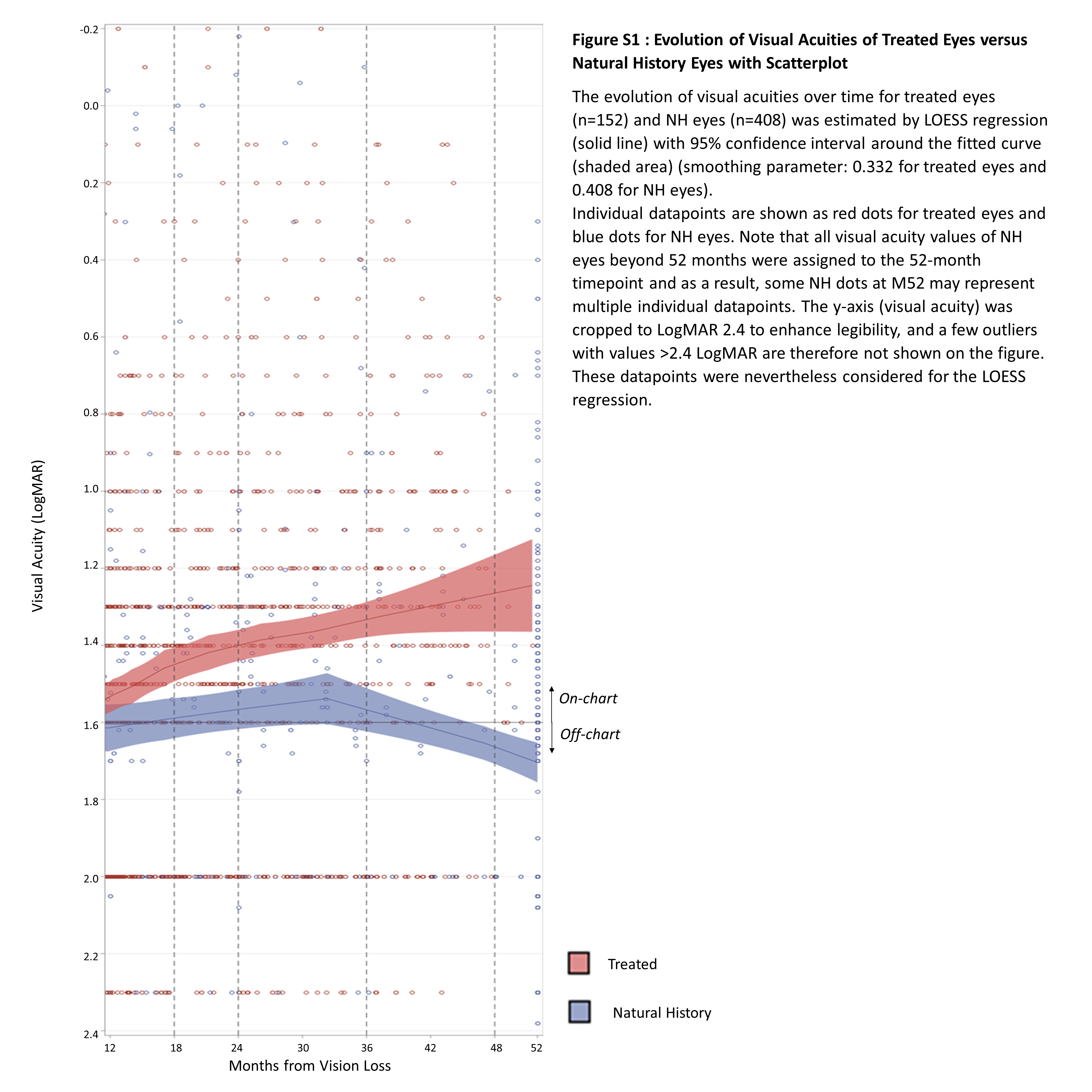

Supplement: Supplementary file 3 [file Image_1.tif]

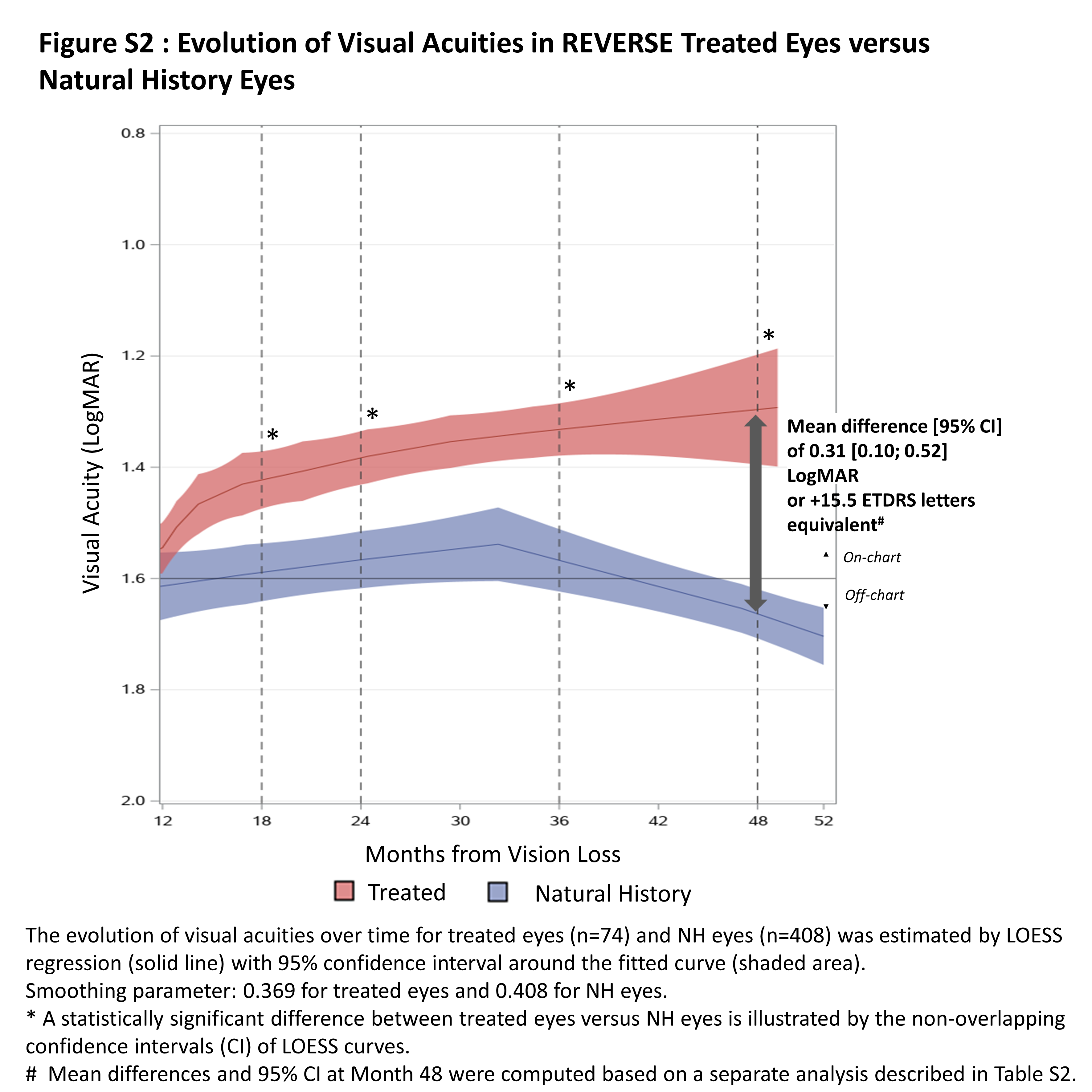

Supplement: Supplementary file 4 [file Image_2.tif]

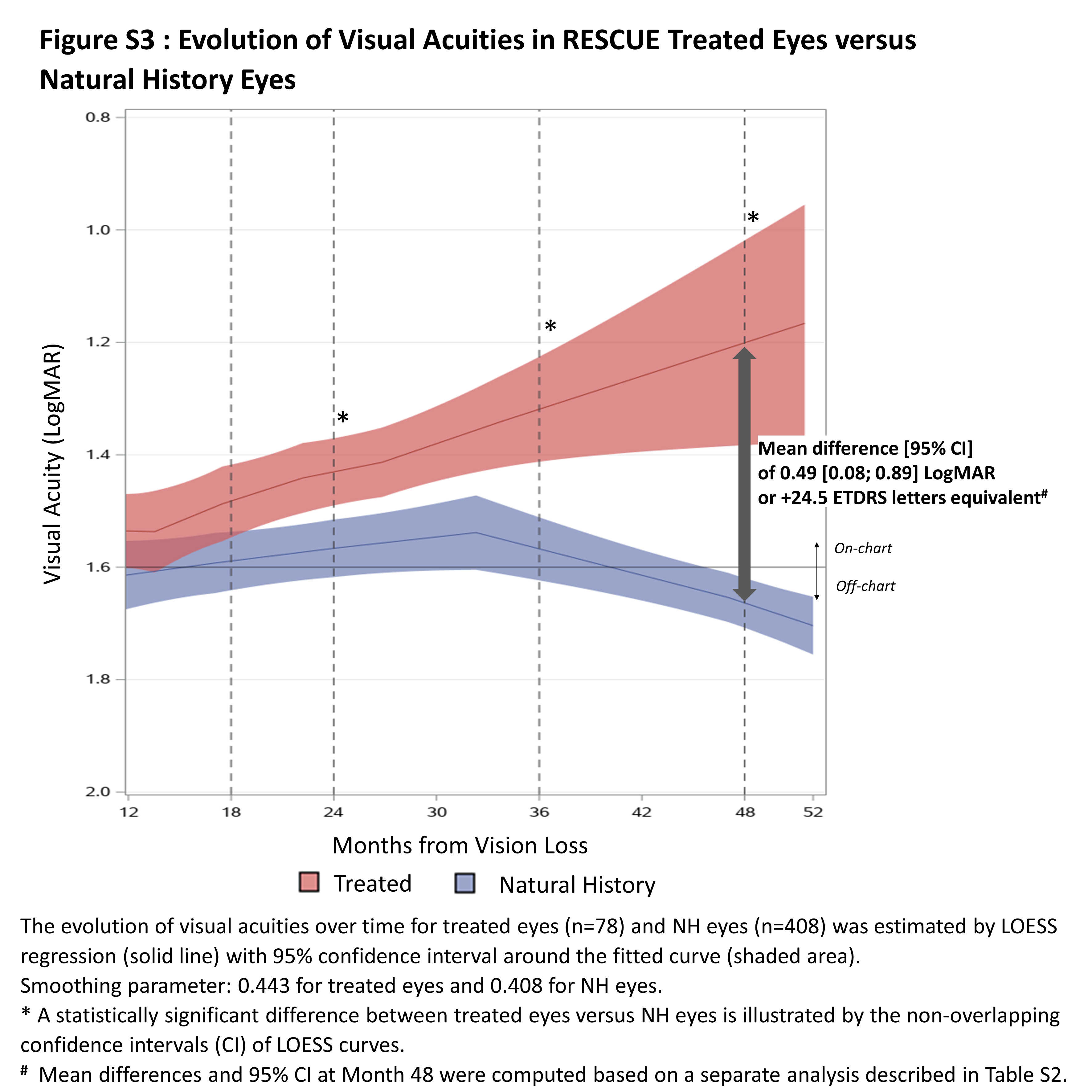

Supplement: Supplementary file 5 [file Image_3.tif]
