# Supplementary material for: Intravitreal Gene Therapy vs. Natural History in Patients With Leber Hereditary Optic Neuropathy Carrying the m.11778G>A ND4 Mutation: Systematic Review and Indirect Comparison
Source: Front Neurol. 2021 May 24;12:662838. doi: 10.3389/fneur.2021.662838 (PMC8181419; doi:10.3389/fneur.2021.662838)
Supplement: Supplementary file 6 [file Table_1.docx]

**Table S1: Studies and Patients Included in the Indirect Comparison Analysis**

| **Study ID** | **Type of Visual Acuity Data^a^** | **Number of Patients with the** **m.11778G>A *ND4* Mutation** | **Number of Patients Included in the Analysis^b^** |
| --- | --- | --- | --- |
| **LHON Patients Treated with** **rAAV2/2-*ND4***^c^ | | | |
| REVERSE | Longitudinal | 37 | 37 |
| RESCUE | Longitudinal | 39 | 39 |
| Extension study of REVERSE and RESCUE | Longitudinal | 62 | 62 |
| ***Total patients in the pooled treated group*** | | ***76*** | ***76*** |
| **Natural History LHON Patients** | | | |
| REALITY | Longitudinal | 44 | 23 |
| Hotta 1995 (20) | Cross-sectional | 89 | 32 |
| Lam 2014 (19)^d^ | Longitudinal | 44 | 36 |
| Nakamura 1993 (21) | Cross-sectional | 9 | 9 |
| Newman 1991 (3) | Cross-sectional | 56 | 40 |
| Qu 2007 (22) | Cross-sectional | 10 | 7 |
| Qu 2009 (23) | Cross-sectional | 14 | 12 |
| Romero 2014 (24) | Cross-sectional | 21 | 15 |
| Sadun 2004 (25) | Cross-sectional | 20 | 14 |
| Yang 2016 (26) | Longitudinal | 16 | 5 |
| Zhou 2010 (27) | Cross-sectional | 25 | 15 |
| ***Total patients in the pooled external control group*** | | ***348*** | ***208*** |

LHON = Leber Hereditary Optic Neuropathy; *ND4* = gene coding for NADH dehydrogenase 4.

a. Visual Acuity data were either longitudinal (several measurements over time per patient) or cross-sectional (measurement at a single point in time per patient).

b. LHON patients with the m.11778G>A *ND4* mutation who were 15 years or older at onset of vision loss and who had Visual Acuity values with reported time of measurement since vision loss.

c. Patients received a single intravitreal injection of rAAV2/2-*ND4* in REVERSE and RESCUE and were followed-up in a joint long-term extension study.

d. Individual patient data were extracted from the poster: Guy J, Feuer W, Davis JL, et al. Gene Therapy for Leber Hereditary Optic NeuropathY An update of Where We Stand (Clinicaltrials.gov number: NCT02161380). Invest Ophthalmol Vis Sci. 2019;60(9):3602.].
