# Supplementary material for: Intravitreal Gene Therapy vs. Natural History in Patients With Leber Hereditary Optic Neuropathy Carrying the m.11778G>A ND4 Mutation: Systematic Review and Indirect Comparison
Source: Front Neurol. 2021 May 24;12:662838. doi: 10.3389/fneur.2021.662838 (PMC8181419; doi:10.3389/fneur.2021.662838)
Supplement: Supplementary file 7 [file Table_2.docx]

**Table S2: Visual Acuity in REVERSE and RESCUE: Treated Eyes versus Natural History Eyes with time intervals from vision loss**

| **Time from Vision Loss** | **Treated** | | **Natural History**  **(N=408 eyes)** |
| --- | --- | --- | --- |
|  | **REVERSE**  **(N=74 eyes)** | **RESCUE**  **(N=78 eyes)** |  |
| **Month 12 - [9;15[ months** |  |  |  |
| Number of eyes | 74 | 76 | 76 |
| Visual Acuity (LogMAR) |  |  |  |
| Median | 1.50 | 1.60 | 1.70 |
| Mean (SD) | 1.55 (0.44) ^#^ | 1.60 (0.64) | 1.69 (0.67) |
| 95% CI (mean) | [1.44;1.65] | [1.45;1.74] | [1.54;1.84] |
| **Month 18 - [15;21[ months** |  |  |  |
| Number of eyes | 74 | 75 | 57 |
| Visual Acuity (LogMAR) |  |  |  |
| Median | 1.40 | 1.40 | 1.60 |
| Mean (SD) | 1.41 (0.41) ^##,^ * | 1.51 (0.60) | 1.60 (0.54) |
| 95% CI (mean) | [1.32;1.50] | [1.37;1.64] | [1.46;1.75] |
| **Month 24 - [21;30[ months** |  |  |  |
| Number of eyes | 74 | 72 | 80 |
| Visual Acuity (LogMAR) |  |  |  |
| Median | 1.30 | 1.40 | 1.52 |
| Mean (SD) | 1.34 (0.45) ^##,^ * | 1.46 (0.70) | 1.54 (0.52) |
| 95% CI (mean) | [1.24;1.45] | [1.29;1.62] | [1.42;1.65] |
| **Month 36 - [30;42[ months** |  |  |  |
| Number of eyes | 70 | 58 | 66 |
| Visual Acuity (LogMAR) |  |  |  |
| Median | 1.30 | 1.40 | 1.55 |
| Mean (SD) | 1.29 (0.48) ^##,^ ** | 1.38 (0.69) | 1.52 (0.47) |
| 95% CI (mean) | [1.18;1.41] | [1.20;1.56] | [1.40;1.63] |
| **Month 48 - [42;54[ months** |  |  |  |
| Number of eyes | 56 | 6 | 27 |
| Visual Acuity (LogMAR) |  |  |  |
| Median | 1.30 | 1.15 | 1.62 |
| Mean (SD) | 1.27 (0.45) ^##,^ ** | 1.10 (0.40) ^#,^ * | 1.59 (0.44) |
| 95% CI (mean) | [1.15;1.39] | [0.68;1.52] | [1.41;1.76] |

CI = confidence interval; LogMAR = logarithm of the minimal angle of resolution; SD = standard deviation.

The time from vison loss was calculated for each eye of each patient. For each eye, only the closest value to the nominal timepoint was selected based on the time windows indicated in brackets.

^#^ P <0.05, ^##^ P <0.01: statistically significant differences versus NH eyes using Kruskal-Wallis test.

* P <0.05, ** P <0.01: statistically significant difference versus NH eyes using mixed ANCOVA
